# A pH-Indicating Colorimetric Tough Hydrogel Patch towards Applications in a Substrate for Smart Wound Dressings

**DOI:** 10.3390/polym9110558

**Published:** 2017-10-26

**Authors:** Li Liu, Xinda Li, Masanori Nagao, Anastasia L. Elias, Ravin Narain, Hyun-Joong Chung

**Affiliations:** 1 Department of Chemical and Materials Engineering, University of Alberta, 116 Street and 85 Avenue, Edmonton, AB T6G 2V4, Canada; lliu3@ualberta.ca (L.L.); xinda1@ualberta.ca (X.L.); nanonanounivers@gmail.com (M.N.); aelias@ualberta.ca (A.L.E.); narain@ualberta.ca (R.N.); 2 Department of Chemical Engineering, Kyushu University, 744 Motooka, Nishi-ku, Fukuoka 819-0395, Japan

**Keywords:** smart hydrogels, pH responsive dye, molecular modification, mechanical testing, stretchable substrates

## Abstract

The physiological milieu of healthy skin is slightly acidic, with a pH value between 4 and 6, whereas for skin with chronic or infected wounds, the pH value is above 7.3. As testing pH value is an effective way to monitor the status of wounds, a novel smart hydrogel wound patch incorporating modified pH indicator dyes was developed in this study. Phenol red (PR), the dye molecule, was successfully modified with methacrylate (MA) to allow a copolymerization with the alginate/polyacrylamide (PAAm) hydrogel matrix. This covalent attachment prevented the dye from leaching out of the matrix. The prepared pH-responsive hydrogel patch exhibited a porous internal structure, excellent mechanical property, and high swelling ratio, as well as an appropriate water vapour transmission rate. Mechanical responses of alginate/P(AAm-MAPR) hydrogel patches under different calcium and water contents were also investigated to consider the case of exudate accumulation into hydrogels. Results showed that increased calcium amount and reduced water content significantly improved the Young’s modulus and elongation at break of the hydrogels. These characteristics indicated the suitability of hydrogels as wound dressing materials. When pH increased, the color of the hydrogel patches underwent a transition from yellow (pH 5, 6 and 7) to orange (7.4 and 8), and finally to red (pH 9). This range of color change matches the clinically-meaningful pH range of chronic or infected wounds. Therefore, our developed hydrogels could be applied as promising wound dressing materials to monitor the wound healing process by a simple colorimetric display, thus providing a desirable substrate for printed electronics for smart wound dressing.

## 1. Introduction

Wound healing is a complex regeneration process which requires collaborative efforts of many different tissues and cell lineages [1,2]. The pH value within a wound milieu is an important parameter for therapeutic interventions in wound care because it both reflects and influences numerous fundamental physiological and biochemical processes evolved in wound remodeling [3]. Under normal circumstances the skin surface is acidic [4,5] with varied pH from 4 to 6, which supports the skin’s natural barrier function and helps to counteract microbial colonisation [6,7]. However, this acidic milieu is easily affected by wounds that cause a fluidic mixing with body’s internal fluid, whose pH value is 7.4. Clinical investigations have demonstrated that the pH of chronic and infected wounds exist in the range of 7.5–8.9, which is an alkaline environment [8,9]. Within this alkaline condition, the healing progression is decreased when compared to the wounds with a pH close to neutral [10,11]. More recent research showed further that a chronic wound is in an alkaline state before the healing process; it progresses to a neutral, and then an acidic state as healing evolves. Therefore, pH could be used as an indicator of wound healing. Considering the importance of the wound pH, monitoring the surface pH in wounds could be helpful in guiding management practices and in determining effective treatment strategies [12].

Several platforms [13,14,15] have been proposed in recent years to monitor pH in wounds. Wang et al. [13] described a wearable potentiometric pH cell embedded in an adhesive bandage to sense the pH changes of wounds. An optical fiber sensor for monitoring pH reversibly and in real-time, based on a porous sol-gel layer doped with bromophenol blue dye, has also been recently reported [14]. Sridhar and Takahata [15] developed a hydrogel-based passive wireless sensor with a micromachined transducer to continuously track wound pH. As the hydrogel swells and deswells with pH, the distance between two planar coils can be converted to inductance. These systems for wound pH sensing are based on either electrochemical or colorimetric methods. The electrochemical technique provides high accuracy and sensitivity, however, the sensors suffer from degradation of performances stemming from mechanical fragility, fouling by non-specific absorption, and requirement of frequent recalibration [16]. The other colorimetric method usually exploits indicator dyes, which are immobilized on the surface of sensors by covalent linkage or physical entrapment. It has advantages of high flexibility, toughness and easily visible signals [17]. As a drawback, many of them are easily leached from the matrix, thus making long-term use and wound application impractical [18].

Hydrogels are water-swollen polymeric materials that maintain a distinct three-dimensional structure [19]. They have been explored for a variety of biological applications, such as regenerative medicine, drug delivery, stem cell and cancer research, as well as wound dressing, due to their tunable physical, chemical, and biological properties, high biocompatibility, ease of fabrication, and similarity to native extracellular matrix. However, applications of hydrogels in medicine have traditionally been limited to those which do not require physical integrity as hydrogels’ mechanical strength is usually weak [20,21,22]. The alginate/polyacrylamide (PAAm) double network hydrogel exhibits excellent mechanical strength and biocompatibility. It possesses an ionically-crosslinked first network Ca-alginate and a covalently-crosslinked second network polyacrylamide. The exceptional toughness of alginate/PAAm can be explained by the fact that the first densely-crosslinked alginate (primary network) provides the hydrogel strength and the second loosely-crosslinked PAAm (secondary network) allows for energy dissipation at large strains [23,24].

Here, we synthesized a pH indicating colorimetric tough hydrogel alginate/P(AAm-MAPR) for smart wound dressing application. The pH indicator dye, phenol red, was modified with a methacryloyl moiety to allow the modified monomer to participate in the radical polymerization reaction of PAAm. Unattached dye molecules, which can potentially be cytotoxic, were removed by dialysis. The structure of the modified phenol red was verified by proton nuclear magnetic resonance (^1^H NMR) and Fourier transform infrared (FTIR) spectroscopy. Then, a series of hydrogels were crosslinked by varying wt % of crosslinking agents (*N,N*′-methylenebisacrylamide and calcium ion). Physical properties of the resulting materials were characterized, including color changes, surface and internal morphologies, mechanical property, swelling ratio, and water vapour transmission rate. We found that our prepared alginate/P(MAPR-AAM) hydrogel patches were flexible, moist, mechanically strong, and able to indicate the pH level of immersing solution by a simple color observation, which are desirable traits as a substrate for smart wound dressing.

## 2. Materials and Methods

### 2.1. Materials

Phenol red dye, methacryloyl chloride, triethylamine (TEA), anhydrous tetrahydrofuran (THF), dimethyl sulfoxide (DMSO), DMSO-*d*_6_, ammonium persulphate (APS), *N,N’*-methylenebisacrylamide (MBAA), acrylamide (AAm), *N,N,N*′*,N*′-tetramethylethylenediamine (TEMED), calcium chloride (CaCl_2_), and sodium alginate were purchased from Sigma-Aldrich (Oakville, ON, Canada)and used as received. Potassium sodium buffer solutions with pH values of 5, 6, 7, 7.4, 8, and 9 were obtained from Fisher Scientific (Ottawa, ON, Canada). All other chemicals used in this work were used as received.

### 2.2. Synthesis and Characterization of Methacrylated Phenol Red (MA-PR)

Phenol red (0.5 g, 0.0014 mol) and triethylamine (250 µL, 0.0018 mol) were dissolved in anhydrous THF (40 mL). The solution was kept in an ice bath. Afterwards, a mixture of methacryloyl chloride (136.8 µL, 0.0014 mol) and anhydrous THF (10 mL) was added dropwise under a nitrogen atmosphere, and the resulting solution was stirred overnight. The reaction mixture was filtered and THF was removed by rotary evaporation resulting in red dye powders, which were reserved for the subsequent preparation of smart hydrogel substrates.

In order to characterize the chemical structure of MA-PR, DMSO was selected as the solvent to synthesize MA-PR. Similarly, phenol red (0.15 g, 0.0004 mol) and triethylamine (83 µL, 0.0005 mol) were dissolved in anhydrous DMSO (4 mL). Methacryloyl chloride (41.04 mL, 0.0004 mol) and DMSO (1 mL) were then added dropwise to the above solution and stirred overnight. The reaction mixture was filtered and DMSO was removed by freeze drying at −40 °C for 8 h. The resultant was vacuum-dried at 70 °C to obtain red modified dye powders MA-PR. The chemical structure of MA-PR was confirmed by Fourier transform infrared spectroscopy (Nicolet 8700, Nicolet Instrument Co., Madison, WI, USA). To obtain FTIR spectra, the dye powders were mixed with dry KBr powders (Sigma-Aldrich, Oakville, ON, Canada) and then loaded into the sample holder. The resolution was 2 cm^−1^ and the sample was scanned from 4000 to 400 cm^−1^ for 64 times. The ^1^H NMR spectra was recorded on a VNMRS 600 MHz spectrometer (Varian Inc., Palo Alto, CA, USA) with DMSO-*d*_6_ being the solvent. Chemical shifts were recorded in ppm and referenced against tetramethylsilane (TMS). MA-PR powders were dissolved in buffer solutions from pH 5 to pH 9 to observe the color changes and then characterized by UV-VIS spectroscopy. The cuvette with MA-PR buffer solution was placed in the sample compartment of the UV-VIS spectrophotometer (Perkin-Elmer NIR-UV, PerkinElmer, Waltham, MA, USA) and the UV-VIS spectrum was recorded.

### 2.3. Preparation of P(AAm-MAPR)/Alginate Hydrogel Patches

The hybrid hydrogel was synthesised by two steps following to the method developed by Zhou et al. [24]. Firstly, sodium alginate (0.1 g, 12.5 wt % to AAm), modified dye MA-PR (0.001 g, 0.125 wt % to AAm), and AAm grain (0.8 g) were dissolved in deionized water (5 mL). MBAA (0.0008 g, 0.1 wt % to AAm), as the crosslinking agent of MA-PR and AAm, and APS (0.0008 g, 0.1 wt % to AAm), as the thermo-initiator, were subsequently added into the solution. The mixture was stirred to become a homogeneous solution and then degassed under vacuum for 20 min to eliminate any entrapped air. After adding the accelerator TEMED (5 µL), the solution was transferred into a glass mould and incubated in an oven at 70 °C for 3 h. This step produced a Na-alginate/P(AAm-MAPR) hydrogel. Secondly, the Na-alginate/P(AAm-MAPR) hydrogel was soaked in a 2 wt % CaCl_2_ solution (0.2 g CaCl_2_ in 10 mL H_2_O) for 3 h at room temperature to crosslink the alginate. Afterwards, the synthesized P(AAm-MAPR)/alginate hydrogel patch was dialysed in deionized water for three days to wash away unreacted monomers, and then dried in a vacuum oven at 65 °C for 2 h. The blank hydrogel without MA-PR dye was also synthesized as the control group using the same recipe.

A series of P(AAm-MAPR)/alginate hydrogels with 0.05 wt % MBAA (0.0004 g), 0.1 wt % MBAA (0.0008 g), and 0.15 wt % MBAA (0.0012 g) were prepared. Amounts of other chemicals were kept the same as used in the above recipe. Similarly, hydrogels were also synthesized with varying amounts of CaCl_2_: 0.1 wt % (0.01 g in 10 mL H_2_O), 1 wt % (0.1 g in 10 mL H_2_O), 2 wt % (0.2 g in 10 mL H_2_O), and 5 wt % (0.5 g in 10 mL H_2_O).

### 2.4. Characterization

The morphologies of as-synthesized P(AAm-MAPR)/alginate hydrogels with different concentrations of MBAA were observed with a scanning electron microscope (SEM) (Zeiss EVO MA10, Zeiss, Germany). After the hydrogel was immersed in deionized water for one day, it was rapidly frozen in liquid nitrogen at −40 °C and lyophilized at −50 °C for 24 h in a SuperModulyo freeze dryer (Thermo Fisher Scientific, Waltham, MA, USA). The swollen freeze-dried sample was then mounted onto an aluminum stub and sputter-coated with gold for SEM observation. P(AAm-MAPR)/alginate hydrogels were soaked in buffer solutions with pH values from 5 to 9 to observe the color changes, and then characterized by UV-VIS spectroscopy. The buffer solution-soaked hydrogels were cut into strips to fit in the cuvette and subsequently placed in the sample compartment of the UV-VIS spectrophotometer (Perkin-Elmer NIR-UV, PerkinElmer, Waltham, MA, USA). UV-VIS spectra were recorded and analyzed.

### 2.5. Mechanical Testing

The mechanical properties of the prepared hydrogel patches with various water contents and crosslinking densities were measured using an Instron 5943 tensile tester (Instron, Norwood, MA, USA) equipped with a 10 kN load cell at room temperature. Hydrogels were cut into strips with dimension of 6 cm × 1 cm × 0.1 cm. Each end of the sample was gripped and stretched with a crosshead speed of 100 mm/min. The Young’s modulus (E) was measured from the slope of the linear section of the stress-strain curve. The elongation at break was calculated by comparing the length of the hydrogel at the breakage to its initial length. Six measurements were repeated for each sample to obtain the average value.

### 2.6. Swelling Ratio Test

Hydrogel patch samples with three MBAA concentrations (0.05 wt %, 0.1 wt %, and 0.15 wt %) were cut into 2 cm × 2 cm squares and dried at 65 °C under vacuum for 24 h. Subsequently, hydrogel squares were immersed in phosphate buffer solution (PBS, pH 7.4) at 37 °C for 72 h until equilibrium. At specific time intervals samples were removed from solutions, and excessive surface water was dried with filter paper. The weights of samples were measured. All measurements were conducted three times to confirm the values. The swelling ratio (*SR*) was determined according to Equation (1):

(1) $$SR\%=\left(W_{s}-W_{d}\right)/W_{d}\times 100$$

where *W*_s_ and *W*_d_ are the weights of the hydrogel sample swollen in PBS at 37 °C and dried for 24 h at 65 °C, respectively.

### 2.7. Water Vapour Transmission Test

The water vapour transmission rates (WVTR) of hydrogel patches with three MBAA concentrations (0.05 wt %, 0.1 wt %, and 0.15 wt %) were determined according to the ASTM E96 standard method. Briefly, the hydrogel patches were mounted and sealed on the top of opened vials (diameter of 17 mm) containing deionized water (20 mL). Barrier tapes were used to tightly fasten the hydrogel patches. The hydrogel-vial assemblies were placed in an isothermal incubator at 37 °C for 72 h. Periodic weightings were carried out to measure the water evaporation through the hydrogel patches. The measurement was repeated three times for each sample. The WVTR was determined by dividing the daily weight loss of water with the area of the vial opening.

## 3. Results and Discussion

### 3.1. Synthesis and Characterization of MA-PR

**Figure 1:**
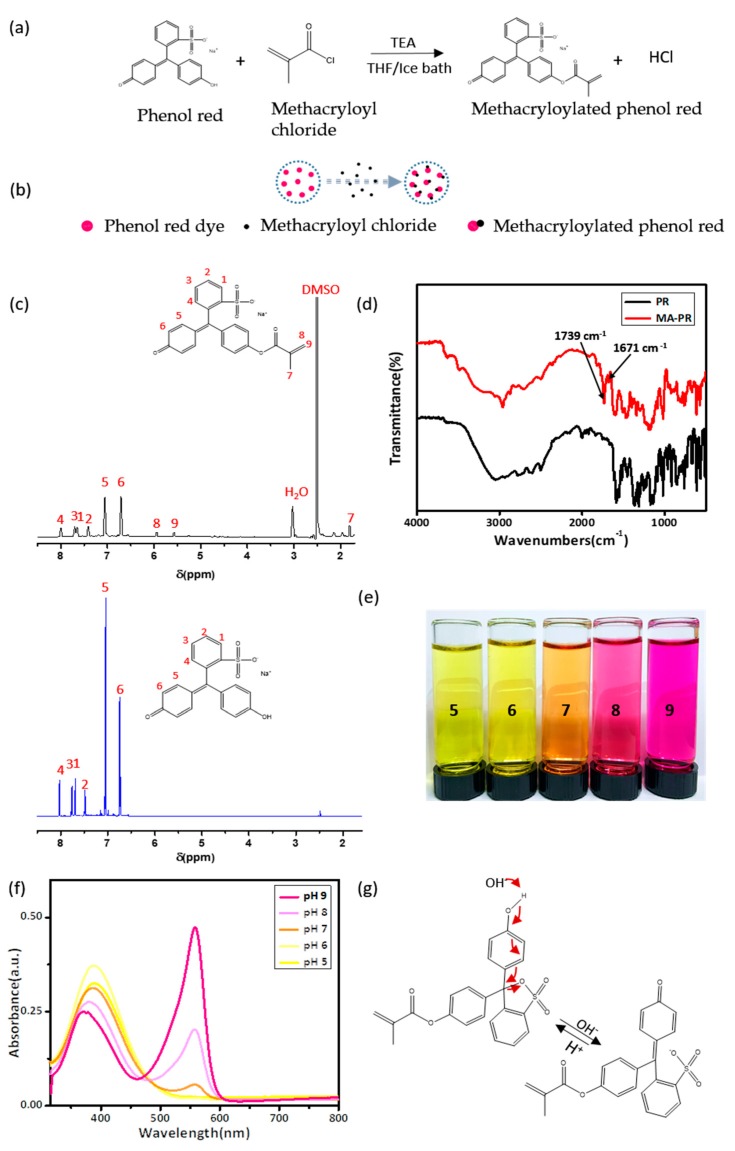
Synthesis and characterization of MA-PR. (**a**) Reaction scheme for the preparation of MA-PR; (**b**) schematic diagram for the synthesis of MA-PR; (**c**) ^1^H NMR spectra of PR (blue) and MA-PR (black); (**d**) FTIR spectra of PR and MA-PR; (**e**) a photographic image that shows the colorimetric transition of MA-PR in buffer solutions with pH values from 5 to 9 (from left to right); (**f**) UV-VIS absorption spectra of MA-PR in buffer solutions with pH values from 5 to 9; and (**g**) a schematic drawing that represents the resonance transition in the MA-PR molecule in acidic (left) and basic (right) environments.

The pH indicator dye, phenol red (PR), consists of three benzene rings with one sulfonate group and two hydroxyl groups that render pH-sensitivity. Nucleophilic substitution of one hydroxyl group on a PR dye molecule with methacryloyl chloride results in the formation of a methacrylated dye, MA-PR [25,26,27,28]. The reaction scheme and the concept of the functionalization are depicted in Figure 1a,b, respectively. This one-step modification was simple and straightforward to perform. To prepare MA-PR for the subsequent synthesis of hydrogel patch, THF was used as the solvent due to its low boiling point. After the reaction, THF could be easily removed by rotary evaporation. The resultant crude product was a mixture of both modified MA-PR and unreacted PR molecules, and would participate in the following copolymerization with AAm for obtaining hydrogel patches. Purification was, thus, not necessary as unattached PR molecules would be washed away by the dialysis.

Phenol red is a stubborn material and cannot be completely dissolved by most common solvents, including THF. In order to obtain the pure MA-PR molecules, DMSO was selected as the reaction solvent due to its high solubility with PR dyes. The excess of methacryloyl chloride would consume all PR molecules resulting in pure MA-PR, as this modification was known to be highly efficient. DMSO was subsequently removed by freeze-drying. FTIR and ^1^H NMR spectroscopies were used to reveal the chemical structure of synthesized MA-PR. ^1^H NMR spectroscopy is illustrated in Figure 1c; here the spectrum of PR was compared with that of the MA-PR. The peak at 1.81 ppm (7) was assigned to the protons of CH_3_ on methacrylate. The two singlets at 5.57 (9) and 5.94 ppm (8) were assigned to the vinyl protons of CH_3_–C=CH_2_*, the splitting could be attributed to the conjugation of carbonyl and vinyl group. This conjugation limited the mobility of double bonding and, consequently, the two protons on the vinyl group experienced different chemical environments to each other. These peaks (7, 8, and 9) were associated with the methacrylate group in MA-PR as they showed shifts from the 6.05 and 6.51 ppm on methacryloyl chloride structure [25]. The multiple peaks in the range of 6.74–8.02 ppm were assigned to the protons in the aromatic rings. In addition to the characteristic peaks of MA, some solvent peaks also existed on the spectrum of MA-PR, such as DMSO and H_2_O peaks. The results of ^1^H NMR indicated that unsaturated C=C bonds were successfully introduced to PR in the MA-PR.

FTIR spectra of PR and MA-PR were compared in Figure 1d. PR and MA-PR both exhibited a phenol band consisting of two components: a broad band centered at 2967 cm^−1^, attributed to hydrogen-bonded phenol groups, and a relatively narrow band at 3584 cm^−1^, assigned to free phenol groups. The typical stretching absorption of carbonyl group in the methacrylate structure was located at 1739 cm^−1^; only MA-PR spectrum exhibited the peak. The peak at 1671 cm^−1^ originated from the C=C stretching of the vinyl structure and multiple peaks from 1460–1640 cm^−1^ were due to the C=C stretching of aromatic rings. Bands at 1156 and 1366 cm^−1^ were resulted from stretching vibrations of sulfonate groups. In summary, the results of ^1^H NMR and FTIR confirmed the modification of MA-PR dye was as we designed.

MA-PR dyes displayed a distinct color variation in buffer solutions with five different pH values. With increasing pH, the MA-PR dyes underwent a transition from yellow (pH 5) to orange (pH 7) and, finally, to red (pH 9), as shown in Figure 1e. The color changes were quantitatively displayed in the UV-VIS spectra as a function of pH. In Figure 1f, the MA-PR showed maximum absorbance peaks at 388 nm in acidic conditions. The peak intensity decreased as pH became more basic, and a peak at 588 nm emerged. The peak at 588 nm corresponded to the deprotonated form of the dyes, which became abundant as pH increased. The transition in the dominant peak was due to the resonance transformation in the molecular structure of MA-PR, as schematically described in Figure 1g. The peak at 388 nm was attributed to the π-π* transition of the conjugated benzenoid ring system. When the solution became more alkaline, the new absorption band at 558 nm was assigned to the n-π* transition resulting from benzenoid to quinoid excitonic transformation.

### 3.2. Preparation and Characterization of P(AAm-MAPR)/Alginate Hydrogel Patches

**Figure 2:**
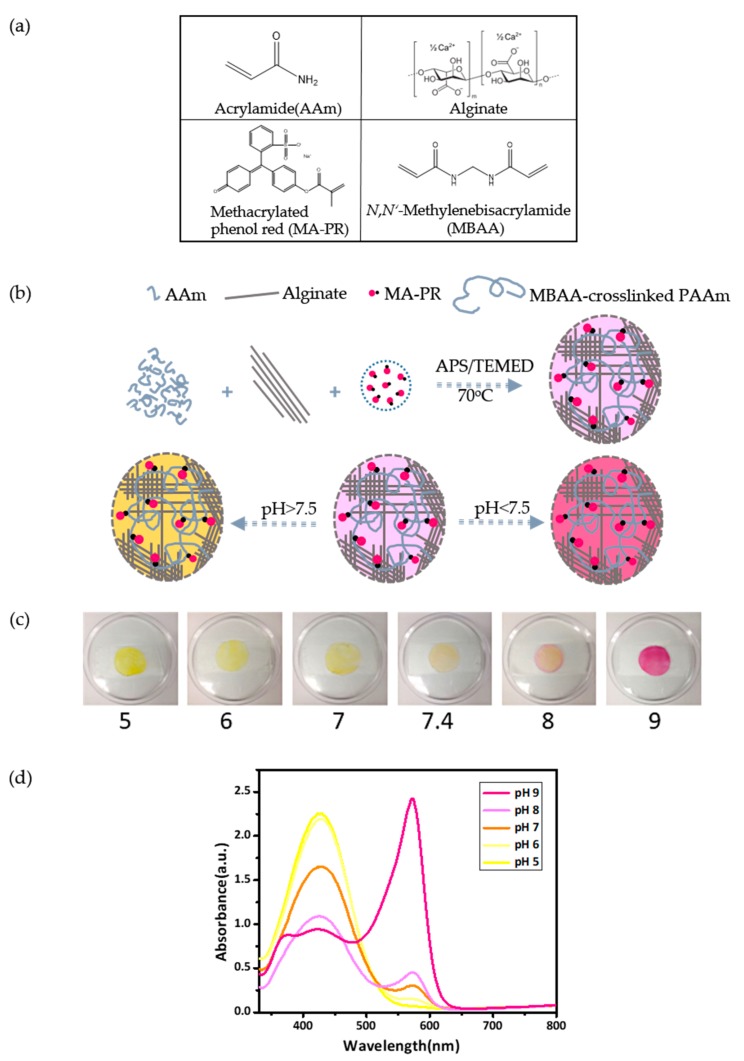
Preparation and characterization of P(AAm-MAPR)/alginate hydrogel patches. (**a**) The chemical structures and the names of the monomers, the dye and the crosslinker used in the synthesis of the alginate/P(AAm-MAPR) hydrogel patch; (**b**) synthetic strategy and colorimetric transitions of the alginate/P(AAm-MAPR) hydrogel patch; and (**c**) photographic images that captured the colorimetric transition of the hydrogel patch in buffer solutions with pH values from 5 to 9 (from left to right). (**d**) UV-VIS absorption spectra of the hydrogel patch while immersed in buffer solutions with pH values from 5 to 9.

The P(AAm-MAPR)/alginate hydrogel patch was synthesized by a two-step approach including the synthesis of MA-PR dye and the synthesis of the double network hydrogel. To fabricate the hydrogel patch, all ingredients were mixed in water to form the two interpenetrating hydrogel networks: sodium alginate and crosslinker CaCl_2_ for the ionically-crosslinked alginate; monomer AAm and MA-PR, crosslinker MBAA, and thermoinitiator TEMED for the covalently-crosslinked P(AAm-MAPR). The chemical structures and names of the monomers, the dye, and the crosslinker are presented in Figure 2a. As a result, the loosely-crosslinked long PAAm polymer chains interpenetrated with the densely-crosslinked Ca-alginate to develop a tough double network hydrogel [24]. A schematic diagram is shown in Figure 2b. The high mechanical strength of this hydrogel could be described as follows [29]: when the load was applied, the loose PAAm network remained intact and stabilized the deformation; meanwhile, alginate networks unzipped progressively. The closely spaced ionic crosslinks unzipped with a small stretch, but the ionic bonding was reversible and non-specific. Therefore, the unzipping of ionic bonding dissipated the fracture energy in an efficient way. After preparation, all P(AAm-MAPR)/alginate hydrogel patches were dialysed for three days to wash away unreacted monomers and dried in a vacuum oven at 65 °C for 2 h; hydrogels in this state were defined as “original state”. Here the swelling ratio of the as-prepared sample (including the dialysis step) with medium crosslinking density of MBAA was measured to be 251.5%.

A transparent medical tape was adhered on one side of the pH-responsive hydrogel patch to create a wearable platform that could be applied to a wound. Subsequently, the patch was soaked in buffer solutions with various pH values (pH = 5, 6, 7, 7.4, 8, 9). The color of the hydrogel patch underwent a transition from yellow (pH 5, 6 and 7) to orange (7.4 and 8), and finally to red (pH 9), as captured by a camera in Figure 2c. The pH window of the color transition matches the pH range that is required to display the status of chronic or infected wounds. As the wound heals, its chronic environment progresses from an alkaline condition to a neutral and then an acidic condition [12]; the hydrogel patch turns from red to orange and then to yellow accordingly. Figure 2d illustrates the UV-VIS absorbance spectra of the MA-PR in the hydrogel patch after exposed to different pH buffer solutions. In acidic buffer, only one absorption peak was observed at 427 nm, similar to the case of the free MA-PR dye in buffer solutions. When the pH varied from acidic to basic, the absorption started to undergo a red shift, as qualified by the decreasing intensity of the peak at 427 nm and by the increasing intensity of peak at 573 nm, which was the result of the resonance transition. A quantitative comparison of the MA-PR peaks between the free and the hydrogel states showed that the most hypsochromic maximum wavelength shifted from 388 nm (free) to 427 nm (hydrogel), whereas the most bathochromic wavelength shifted from 558 nm (free) to 573 nm (hydrogel). A possible explanation was that interactions between the dye and the hydrogel backbone lowered the excitation energy of MA-PR molecules when they were covalently immobilized within hydrogel networks.

### 3.3. Physical Evaluation of Hydrogel Patches as a Function of P(AAm-MAPR) Crosslinking Density

**Figure 3:**
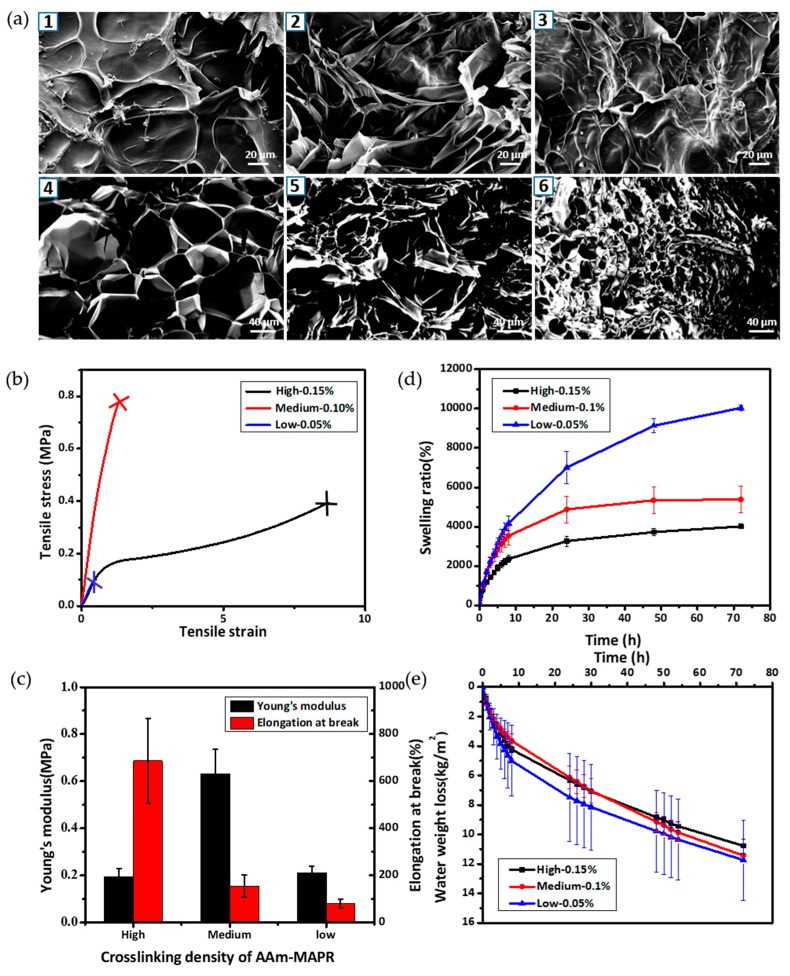
Physical evaluation of hydrogel patches at different MBAA concentrations. (**a**) SEM images of hydrogel patch with three crosslinking densities from the surface (1–3) and the cross-section views (4–6): 1 and 4: high crosslinking density (0.15% MBAA concentration); 2 and 5: medium crosslinking density (0.1% MBAA concentration); and 3 and 6: low crosslinking density (0.05% MBAA concentration); (**b**) tensile stress-strain curves; (**c**) Young’s modulus and elongation at break; (**d**) swelling ratio; and (**e**) water vapour transmission rate of hydrogel patches with the three MBAA crosslinking densities.

The physical properties of the hydrogel patch, including surface and interior morphologies, mechanical properties, swelling ratio, and water vapour transmission rate, were evaluated for P(AAm-MAPR) hydrogels with various crosslinking densities. Figure 3a showed the surface (1–3) and cross-section (4–6) images of various hydrogels observed by SEM. The surface (1–3) did not show visible open pores, but displayed a relatively smooth structure with tangled strands. A comparison between low (1) and high (3) crosslinking densities revealed that the network structure was denser in the higher crosslinking case, as evidenced by the appearance of increased entanglement of the strands. The interior structures of the hydrogels were visualized in the cross-sectional images (4–6). Here, the hydrogel showed a highly porous honeycomb-like structure, which could be helpful for the supply of oxygen, the absorption of exudates, and the containing of a large amount of water [30]. Increasing the concentration of MBAA (the crosslinker for AAm and MAPR) resulted in the reduction of the averaged pore size; the average diameter of pores decreased from 80 to 40 µm, and finally to 20 µm as the MBAA concentration increased from 0.05% to 0.1% and to 0.15%.

The mechanical properties of the hydrogel patches play a significant role in establishing the patch’s structural integrity during the application, which is a dominant factor to determine the target wound. Hydrogel patches must be able to withstand the applied stress on the wound site, and must have high tolerance to swelling or motion-induced deformation to avoid breakage by exudate absorption and by the patient’s movements. The ductility was quantified by determining the elongation at break [31]. Figure 3b showed the tensile stress-strain curves of hydrogel patches with three crosslinking densities (MBAA) as used in Figure 3a. All hydrogel patches were in the original state. Young’s moduli and elongations at break values were obtained from the Figure 3b and presented in Figure 3c. The results revealed that hydrogel patches with the medium crosslinking density had a maximum Young’s modulus of 0.55 MPa, whereas 0.2 MPa was observed in the high and the low crosslinking density cases. This demonstrated that increases in the chemical crosslinking of P(AAm-MAPR) networks or in the physical entanglement between the two networks did not guarantee the increases in the stiffness or strength of the double network alginate/P(AAm-MAPR) hydrogel. Instead, a right amount of fluidity could optimize the dissipation of the applied stress by the alginate, which was a softer network with reversible ionic bonding. Thus, the optimal condition for the enhanced mechanical properties may appear as a specific crosslinking density, as suggested earlier by Gong et al. [32]. Hence, the medium crosslinking density (0.1 wt % MBAA) provided the optimum condition among three crosslinking densities. The increase of MBAA concentration from 0.05 to 0.15 wt % significantly increased the elongation at break of the hydrogel from 80% to 686%. This was ascribed to the enhanced stretchability of the hydrogel originated from the fact that increased MBAA decreased the necessity of breaking non-reversible covalent bondings in the P(AAm-MAPR) network at a given strain level, resulting in detrimental breakage of the structure at lower elongation.

A moist wound environment is important in modern therapy because it can promote the penetration of the substances that aid in the healing process and protect wounds against bacterial invasion. The moisture in wound dressings also enables a painless dressing removal without damage to the newly formed skin [33]. An ideal wound dressing is supposed to lock the exudate and maintain a proper moisture level during the healing process. Therefore, measurements of the swelling ratio and water vapour transmission rate were performed and presented in Figure 3d,e, respectively. Figure 3d showed that the hydrogel patches with the high and the medium crosslinking densities reached a near-equilibrium swelling after 24 h immersion in buffer solutions with physiological pH of 7.4 at 37 °C, whereas the sample with the low crosslinking density did not show a saturation up to 72 h, which was the longest duration of the current study. The equilibrium swelling ratio was in the range of 4000–5000%. The water absorptivity and equilibrium water content of hydrogel patches increased as the concentration of MBAA decreased, which was due to the decreased contraction stress that stemmed from the intrinsic stiffness of hydrogel networks [34]. The high swelling ability of the hydrogel is preferable for wound dressing because such dressings can effectively protect the wound bed from exudate accumulation, thus reducing the necessity for frequent replacement [35].

Wound dressing should have proper water vapour transmission properties to develop a favorable environment for speedy healing. Therefore, water vapour transmission rate, which is a qualified form of the water vapour diffusion ability, is an important parameter for wound dressing materials [36]. According to the literature, the average WVTRs for normal skin, first degree burns, and granulating wounds are 204 ± 12, 279 ± 26 and 5138 ± 202 g/m^2^/day, respectively [37]. WVTR of the dressing should be neither too low nor too high, since a low permeability may cause the accumulation of the exudates and result in the leakage, whereas a high permeability may lead to excessive dehydration of the wound surface [34]. It has been suggested that the WTVR for a good wound dressing should be in the range of 2000–2500 g/m^2^/day, which can maintain a proper moisture on the wound without dehydration [38]. The WVTR values of the hydrogel patches with three crosslinking densities are shown in Figure 3e. In the first 5 h, the weight loss was rather rapid and the trend was non-linear; linear slopes occurred between 10 and 72 h. The initial non-linearity could be ascribed to the transient period when the vapour molecules condensed on the surface and subsequently solubilized into the porous structure of hydrogels. Once the transient period was over, the later-incoming water vapour molecules experienced a steady-state diffusion through the width of the film [31]. The WVTR was determined from the linear portion of the graph; each of the three hydrogel patches showed similar WVTR values of 2387 g/m^2^/day, belonging to the range of 2000–2500 g/m^2^/day. All the measurements demonstrated that alginate/P(AAm-MAPR) hydrogels prepared in our study were suitable materials for wound dressing applications.

### 3.4. Investigation of Mechanical Properties under Different Calcium and Water Contents

**Figure 4:**
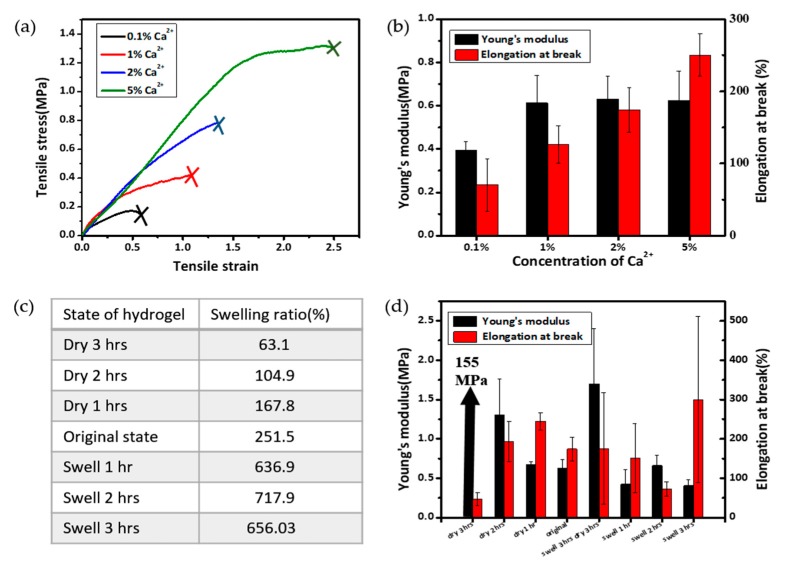
Investigation of the mechanical properties as a function of Ca^2+^ concentrations (**a**,**b**) and time-dependant swelling/drying conditions (**c**,**d**). (**a**) Tensile stress-strain curves and (**b**) Young’s modulus and elongation at break values with various calcium concentrations; (**c**) swelling ratio and (**d**) Young’s modulus and elongation at break values at various time-dependant swelling/drying conditions. All hydrogels have the MBAA concentration of 0.10%. The original state refers to the synthesis condition that hydrogel was vacuum dried in the oven for 2 h at 65 °C after dialysis of three days.

Alginate-based dressings have a pharmacological function due to the action of calcium ions present in the dressing [39]. Calcium released through ion exchange with sodium in the wound exudates can effectively promote hemostasis during the first stage of wound healing [40]. In addition, calcium is the ionic crosslinking agent used in the alginate hydrogel formation, thus, the increase of its content can strengthen the hydrogel’s mechanical strength. Hence, hydrogel patches at various calcium concentrations (0.01%, 0.1%, 0.3% and 0.5%) were prepared to study the effects of calcium concentrations on the mechanical properties. Figure 4a shows the tensile stress-strain curves of the hydrogel patches with various Ca^2+^ concentrations. Similarly, hydrogel patches were in the original state. Figure 4b shows the corresponding values of Young’s moduli and elongations at break. With the increase of Ca^2+^ concentration from 0.01% to 0.1%, the Young’s modulus was moderately increased from 0.39 to 0.61 MPa, whereas the moduli values were nearly invariant at the higher concentration of 0.1–0.5%. It was also found that, from both Figure 4a,b, increasing calcium content resulted in a more stretchable hydrogel. With 5% calcium content, the elongation at break of the hydrogel could be up to 250%. In summary, the Ca^2+^ concentration had a significant influence on the hydrogels’ mechanical properties, especially on the elasticity. For clarification, we note that the Ca^2+^ concentration is kept at 2 wt % unless otherwise specified.

Water content is important for hydrogel dressing because it provides a moist environment for the wound, which is a key factor for the wound healing. It also modulates the permeation of gases and ions to the wound site [41]. However, the swelling of the hydrogel dressing changes with time after the initial application on the wound according to the conditions of wound healing and to the ambient environment. The swelling conditions, especially the water contents in the hydrogel, dictate the mechanical strength of hydrogels. Therefore, we investigated the effect of swelling/drying conditions on the Young’s moduli and elongations at break of our prepared hydrogel patches. Figure 4c shows the various swelling/drying conditions and resulting swelling ratio values. As shown in Figure 4d, the Young’s modulus increased dramatically from 0.67 to 155 MPa as drying time increased from 1 to 3 h. The extremely high mechanical strength of the hydrogel dried for 3 h could be attributed to the exceptionally low water content (i.e., swelling ratio of 63.1%) compared to the that of original patches (251.5%) or other samples. It is notable that the drying experiment mimics the situation when the hydrogel wound dressing is exposed in ambient condition without any encapsulating layer, which is different from the actual application of wound dressing on skin. In the case of swelling up to 3 h, Young’s modulus was in the range of 0.4–0.7 MPa without significant variations. The values of elongation at break showed rather scattered results. However, in general, the elongation at break increased as the swelling ratio increased.

In order to simulate the case that the patch was dried out for some time and then re-swollen from fluid, we tested a sample that dried for three hours and then swelled for three hours. After this treatment, the Young’s modulus was 2.7 times higher than that of the original one, while the elongation remained almost the same. This trend is very different from any of plain drying or plain swelling samples. This may suggest that some irreversible internal structure change happened during the drying process, thus, after three hours of re-swelling the hydrogel did not recover its original modulus.

## 4. Conclusions

A series of pH indicating colorimetric alginate/P(AAm-MAPR) hydrogels were synthesized for potential wound dressing application. The pH indicator dye phenol red was successfully modified with methacrylate and subsequently copolymerized into the double network hydrogel matrix. This covalent attachment could prevent the dye from leaching out of the matrix. When immersed in buffer solutions with various pH values, the color of the hydrogel patch underwent a transition from yellow (pH 5, 6 and 7) to orange (7.4 and 8) and finally to red (pH 9), which matches the required pH range for the application to chronic or infected wounds. The prepared hydrogel patches showed porous internal structures, excellent mechanical properties, high swelling ratio, and appropriate water vapour transmission rates; these features indicated that the hydrogels were suitable for wound dressing applications. In addition, calcium content and water content could significantly impact the Young’s modulus and elongation at break of the hydrogel. In conclusion, our alginate/P(AAm-MAPR) hydrogel could be used as potential wound dressing material that allowed monitoring the wound healing process by a simple colorimetric display. We anticipate this novel substrate to be utilized as a smart and stretchable substrate for printed biomedical electronics.

## References

[1] Carvalho I.C., Mansur H.S. (2017). Engineered 3d-scaffolds of photocrosslinked chitosan-gelatin hydrogel hybrids for chronic wound dressings and regeneration. Mater. Sci. Eng. C.

[2] Martin P. (1997). Wound healing–Aiming for perfect skin regeneration. Science.

[3] Schneider L.A., Korber A., Grabbe S., Dissemond J. (2007). Influence of ph on wound-healing: A new perspective for wound-therapy?. Arch. Dermatol. Res..

[4] Heuss E. (1892). Die Reaktion Des Schweisses Beim gesuNden Menschen.

[5] Schade H., Marchionini A. (1928). Der säuremantel der haut (nach gaskettenmessungen). J. Mol. Med..

[6] Dikstein S., Zlotogorski A. (1993). Measurement of skin ph. Acta Dermato-Venereologica Supplementum.

[7] Rippke F., Schreiner V., Schwanitz H.J. (1999). The acidic milieu of the horny layer/new findings on physiology and pathophysiology of the skin ph-value. Dermatosen in Beruf und Umwelt.

[8] Greener B., Hughes A., Bannister N. The effect of ph on proteolytic activity in chronic wound fluids and methods for determination. Proceedings of the Poster Z079 2nd WUWHS Congress.

[9] Greener B., Hughes A., Bannister N., Douglass J. (2005). Proteases and ph in chronic wounds. J. Wound Care.

[10] Hoffman R., Noble J., Eagle M. (1999). The use of proteases as prognostic markers for the healing of venous leg ulcers. J. Wound Care.

[11] Roberts G., Hammad L., Creevy J., Shearman C., Mani R. Physical changes in dermal tissues around chronic venous ulcers. Proceedings of the 7th European Conference on Advances in Wound Management.

[12] Gethin G. (2007). The significance of surface ph in chronic wounds. Wounds.

[13] Guinovart T., Valdés-Ramírez G., Windmiller J.R., Andrade F.J., Wang J. (2014). Bandage-based wearable potentiometric sensor for monitoring wound ph. Electroanalysis.

[14] Schyrr B., Pasche S., Scolan E., Ischer R., Ferrario D., Porchet J.-A., Voirin G. (2014). Development of a polymer optical fiber ph sensor for on-body monitoring application. Sens. Actuators B Chem..

[15] Sridhar V., Takahata K. (2009). A hydrogel-based passive wireless sensor using a flex-circuit inductive transducer. Sens. Actuators A Phys..

[16] Bergveld P., Sibbald A. (1988). Analytical and Biomedical Applications of Ion-Selective Field-Effect Transistors.

[17] Van der Schueren L., De Clerck K. (2012). Coloration and application of pH-sensitive dyes on textile materials. Color. Technol..

[18] Cooney C.G., Towe B.C. (2000). A miniaturized ph and pco 2 intravascular catheter using optical monitoring and a dual concentric-flow microdialysis approach. Sens. Actuators B Chem..

[19] Ramos M.L.P., González J.A., Fabian L., Pérez C.J., Villanueva M.E., Copello G.J. (2017). Sustainable and smart keratin hydrogel with pH-sensitive swelling and enhanced mechanical properties. Mater. Sci. Eng. C.

[20] Chaturvedi A., Bajpai A.K., Bajpai J., Singh S.K. (2016). Evaluation of poly (vinyl alcohol) based cryogel–zinc oxide nanocomposites for possible applications as wound dressing materials. Mater. Sci. Eng. C.

[21] Khademhosseini A., Langer R. (2007). Microengineered hydrogels for tissue engineering. Biomaterials.

[22] Seliktar D. (2012). Designing cell-compatible hydrogels for biomedical applications. Science.

[23] Peak C.W., Wilker J.J., Schmidt G. (2013). A review on tough and sticky hydrogels. Colloid Polym. Sci..

[24] Yang C.H., Wang M.X., Haider H., Yang J.H., Sun J.Y., Chen Y.M., Zhou J., Suo Z. (2013). Strengthening alginate/polyacrylamide hydrogels using various multivalent cations. ACS Appl. Mater. Interfaces.

[25] Jin L., Agag T., Yagci Y., Ishida H. (2011). Methacryloyl-functional benzoxazine: Photopolymerization and thermally activated polymerization. Macromolecules.

[26] Kandola B.K., Krishnan L., Deli D., Luangtriratana P., Ebdon J.R. (2015). Fire and mechanical properties of a novel free-radically cured phenolic resin based on a methacrylate-functional novolac and of its blends with an unsaturated polyester resin. RSC Adv..

[27] Kermis H.R., Kostov Y., Rao G. (2003). Rapid method for the preparation of a robust optical ph sensor. Analyst.

[28] Lee C.I., Lim J.S., Kim S.H., Suh D.H. (2006). Synthesis and luminescent properties of a novel eu-containing nanoparticle. Polymer.

[29] Sun J.Y., Zhao X., Illeperuma W.R., Chaudhuri O., Oh K.H., Mooney D.J., Vlassak J.J., Suo Z. (2012). Highly stretchable and tough hydrogels. Nature.

[30] Fan Z., Liu B., Wang J., Zhang S., Lin Q., Gong P., Ma L., Yang S. (2014). A novel wound dressing based on Ag/graphene polymer hydrogel: Effectively kill bacteria and accelerate wound healing. Adv. Funct. Mater..

[31] Bajpai S.K., Pathak V., Soni B., Mohan Y.M. (2014). Cnws loaded poly(sa) hydrogels: Effect of high concentration of cnws on water uptake and mechanical properties. Carbohydr. Polym..

[32] Gong J.P., Katsuyama Y., Kurokawa T., Osada Y. (2003). Double-network hydrogels with extremely high mechanical strength. Adv. Mater..

[33] Shezad O., Khan S., Khan T., Park J.K. (2010). Physicochemical and mechanical characterization of bacterial cellulose produced with an excellent productivity in static conditions using a simple fed-batch cultivation strategy. Carbohydr. Polym..

[34] Zheng A., Xue Y., Wei D., Li S., Xiao H., Guan Y. (2014). Synthesis and characterization of antimicrobial polyvinyl pyrrolidone hydrogel as wound dressing. Soft Mater..

[35] Zhang D., Zhou W., Wei B., Wang X., Tang R., Nie J., Wang J. (2015). Carboxyl-modified poly (vinyl alcohol)-crosslinked chitosan hydrogel films for potential wound dressing. Carbohydr. Polym..

[36] Roy N., Saha N., Humpolicek P., Saha P. (2010). Permeability and biocompatibility of novel medicated hydrogel wound dressings. Soft Mater..

[37] Lamke L.-O., Nilsson G., Reithner H. (1977). The evaporative water loss from burns and the water-vapour permeability of grafts and artificial membranes used in the treatment of burns. Burns.

[38] Tsao C.T., Chang C.H., Lin Y.Y., Wu M.F., Wang J.L., Young T.H., Han J.L., Hsieh K.H. (2011). Evaluation of chitosan/γ-poly (glutamic acid) polyelectrolyte complex for wound dressing materials. Carbohydr. Polym..

[39] Boateng J.S., Matthews K.H., Stevens H.N., Eccleston G.M. (2008). Wound healing dressings and drug delivery systems: A review. J. Pharm. Sci..

[40] Lansdown A.B. (2002). Calcium: A potential central regulator in wound healing in the skin. Wound Repair. Regen..

[41] Kamoun E.A., Chen X., Eldin M.S.M., Kenawy E.-R.S. (2015). Crosslinked poly (vinyl alcohol) hydrogels for wound dressing applications: A review of remarkably blended polymers. Arab. J. Chem..

